# Towards psychedelic apprenticeship: Developing a gentle touch for the mediation and validation of psychedelic-induced insights and revelations

**DOI:** 10.1177/13634615221082796

**Published:** 2022-03-22

**Authors:** Christopher Timmermann, Rosalind Watts, David Dupuis

**Affiliations:** 1 Centre for Psychedelic Research, Division of Brain Sciences, Department of Medicine, 4615 Imperial College London, London, UK; 2 Department of Anthropology/Hearing the voice, 3057 Durham University, Durham, UK

**Keywords:** ayahuasca, know-how, neurophenomenology, psilocybin, psychedelic therapy, revelations

## Abstract

A striking feature of psychedelics is their ability to increase attribution of truth and meaningfulness to specific contents and ideas experienced, which may persist long after psychedelic effects have subsided. We propose that processes underlying conferral of meaning and truth in psychedelic experiences may act as a double-edged sword: while these may drive important therapeutic benefits, they also raise important considerations regarding the validation and mediation of knowledge gained during these experiences. Specifically, the ability of psychedelics to induce noetic feelings of revelation may enhance the significance and attribution of reality to specific beliefs, worldviews, and apparent memories which might exacerbate the risk of iatrogenic complications that other psychotherapeutic approaches have historically faced, such as false memory syndrome. These considerations are timely, as the use of psychedelics is becoming increasingly mainstream, in an environment marked by the emergence of strong commercial interest for psychedelic therapy. We elaborate on these ethical challenges via three examples illustrating issues of validation and mediation in therapeutic, neo-shamanic and research contexts involving psychedelic use. Finally, we propose a pragmatic framework to attend to these challenges based on an ethical approach which considers the embeddedness of psychedelic experiences within larger historical and cultural contexts, their intersubjective character and the use of practices which we conceptualise here as forms of *psychedelic apprenticeship*. This notion of *apprenticeship* goes beyond current approaches of *preparation* and *integration* by stressing the central importance of validation practices based on *empathic resonance* by an experienced therapist or guide.

## Introduction

Psychedelics are known for inducing a multifaceted range of effects in human experience, ranging from visual imagery to high-order phenomena such as ego-dissolution and mystical-type experiences (Griffiths et al., 2008; Nour et al., 2016; Preller & Vollenweider, 2016). While research employing psychedelics has generated a growing body of knowledge concerning the psychological and biological mechanisms associated with low-level effects (i.e., visual imagery; Carhart-Harris et al., 2016; de Araujo et al., 2012; Kometer & Vollenweider, 2016), phenomena associated with more abstract (or “high-level”) experiences, such as achieving psychological insights and enhanced attribution of meaning, are less understood. In this article we propose that psychedelic-induced insights and revelations act as a “double-edged sword”: while these revelations may drive important therapeutic benefits and increases in well-being (Carhart-Harris, Roseman, et al., 2018; Davis et al., 2019; Watts et al., 2017), they may also serve as mechanisms for experiences associated with high levels of psychological distress (e.g., troubling revelations). Furthermore, we argue that psychedelic-induced insights may result in significant modifications in worldviews and beliefs, which carries significant bioethical implications. These ethical issues are bound to become increasingly relevant, as the use of psychedelic substances is rapidly achieving mainstream status, especially in the context of the emergence of commercial interest for psychedelic therapy, the so-called psychedelic science “renaissance”, and ritualistic uses of psychedelic substances in secular and neo-shamanic^1^ contexts.

A growing literature has highlighted the central importance that both mystical-type and psychological insight experiences have for improvements in depression, addiction and psychological distress associated with terminal illness, during psychedelic therapy (Belser et al., 2017; Carhart-Harris, Bolstridge, et al., 2018; Davis et al., 2019; Garcia-Romeu et al., 2014; Griffiths et al., 2016; Roseman et al., 2018; Watts et al., 2017). We propose that a common mechanism underlies both of these experiences (i.e., mystical-type effects and psychological insights): the striking subjective feeling of gaining unmediated knowledge and revelations (i.e., their *noetic* quality; see Pahnke & Richards, 1970; Shanon, 2005). While commonly associated with mystical-type experiences, we propose that noetic feelings occur in relation to a wide range of contents of psychedelic experiences, including those pertaining to psychological, spiritual or metaphysical themes. This article reflects on ethical considerations associated with such acts of *knowing* during psychedelic experiences, as the perceived validity of psychedelic experiences may result in significant psychological, social and epistemological issues. We illustrate these issues through three examples of use of psychedelics in different contexts: (1) autobiographical insights observed in a clinical trial using psilocybin for the treatment of depression; (2) neo-shamanic practices in the Peruvian Amazon; and (3) “metaphysical” insights and revelations occurring in naturalistic and controlled research environments. Finally, we outline a framework to navigate the delicate terrain of psychedelic *knowing*, rooted in experiential and embodied ways of *apprenticeship*. Forms of psychedelic apprenticeship may find expression in different instances, such as the opportunities for psychedelic therapists to deepen their therapeutic work, the development of models which highlight the need for nuanced forms of preparation and integration of psychedelic experiences and research which fosters detailed phenomenological inquiry. We propose that forms of *psychedelic apprenticeship* may provide significant benefits to attend to challenges associated with the mediation of psychedelic experiences, especially considering the tension between these issues and the rapidly accelerating interest to develop psychedelic medicines for large populations.

### Mediation of revelations and insights

As mentioned above, we propose that (1) psychedelics are prone to induce insights (or more broadly instances of *knowing*) and (2) the contents of these revelations are broad-ranging; i.e., they may range from biographical events to metaphysical “revelations” (see Grof, 1975; Shanon, 2005; Strassman, 2001, for phenomenological examples). Importantly, the revelatory facet concerning the knowledge experienced during psychedelic states is usually imbued with a sense of authority and heightened validation, which are subjectively felt as intuitive and unmediated (Pahnke & Richards, 1970; Shanon, 2005). Furthermore, the consequences of these experiences may carry over long after the pharmacological effects of the substances have subsided. For example, these experiences may be labelled as one of the top five most meaningful experiences in users’ lives (Griffiths et al., 2011), consistent with neurobiological evidence showing long-term changes in brain function (Barrett et al., 2020) and structure (Bouso et al., 2015; Ly et al., 2018).

While the feelings of veracity associated with specific contents of psychedelic experiences may be felt as unmediated, recent evidence shows that similar feelings can be artificially (i.e., contextually) induced via experimental manipulation with (Preller et al., 2017) and without the administration of psychedelics (Laukkonen et al., 2020). The heightened state of suggestibility induced by psychedelics (Carhart-Harris et al., 2015) provides fertile ground for this quality of certainty to be directed by the context in which these experiences occur. Furthermore, noetic experiences tend to have an unqualified character: information is received and believed without a subjective need of external validation or evidence (hence, they are felt as direct and “unmediated” ways of knowing; James, 1902; and see Shanon, 2005, for a detailed description during psychedelic states). This unmediated character, combined with a wide range of evidence resonant with the notion that psychedelics are able to enhance meaningfulness (Studerus et al., 2010), provides fertile ground for problematic issues associated with validation of the information. Furthermore, the picture becomes even more complex when taking into account the positive or negative epistemic consequences that these experiences may have (see Letheby, 2016, for a nuanced take on epistemic risks and benefits of psychedelic experiences).

**Figure 1.**
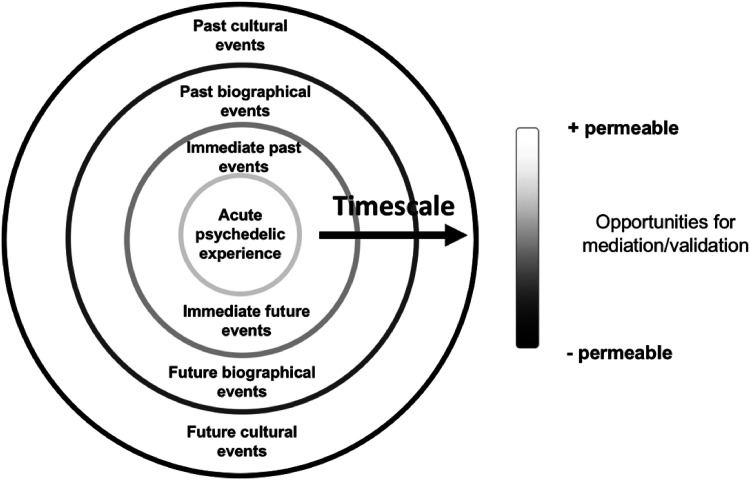
Opportunities for mediation and validation at different temporal scales.

*Note*. A psychedelic experience is embedded in varying temporal scales, which present different opportunities for stronger or weaker forms of mediation. The level of transparency of each of the borders within the concentric circles outlines the degree to which the corresponding instances are permeable for interventions of mediation/validation.

Importantly, while there may be a felt lack of mediation underlying these experiences, forms of mediation are always at play, consistent with evidence stressing the importance of how context influences a psychedelic experience (Carhart-Harris, Roseman, et al., 2018; Metzner et al., 1965). Furthermore, we argue that processes of mediation and validation are not constrained to acute effects experienced in a psychedelic session, but rather extend in time before and after the session. Therefore, forms of mediation and validation of psychedelic insights are subject to broader contexts occurring in wide-ranging temporal scales and are not limited to the acute psychedelic session. Importantly, alterations of levels of suggestibility induced by psychedelics may be conceived as opportunities for forms of mediation and validation at varying temporal scales (Figure 1).

In the following section we will look at three cases as examples of knowledge gained during psychedelic experiences which led us to consider specific issues around the mediation and validation.

## Examples of psychedelic-induced revelations

### Case 1. Biographical revelations in therapeutic contexts

Prevailing models of psychedelic therapy rest on the assumption that psychedelic-induced insights about the self (psychological and biographical information) arise from “intuitive knowing”, or some form of “inner intelligence” (Grof, 1975; Richards, 2016). Often patients will have a strong “felt sense” that their insights are true and real for them, although this is difficult and often unnecessary to prove. However, when an insight relates to biographical revelations, the issue of validity may become very important and preoccupying. For example, a woman with a scar on her hand may receive the insight that her now deceased mother put a cigarette out on her hand when she was an infant. The insight feels true, but whether it is believed holds serious consequences for the woman's life. Such instances are quite frequent in psychedelic therapy, and the way that these insights are “held” by the session facilitators can have important consequences for the patient. Patients primed to believe that everything they see or feel will be “the Truth” may feel preoccupied by any strange or upsetting revelations they might encounter. At the same time, trusting “the inner healing intelligence” is a foundational principle in many models of psychedelic therapy, and the frequent experience of patients and therapists alike is that insights received do hold some important “realness”, and are coming from a place of “deep knowing”. How to hold this space of gentle agnostic respect for the messages that arise is one of the challenges of psychedelic therapy.

We present the case of a participant in a recent psilocybin trial in which a distressing revelation of a biographical event presented issues regarding its validity (i.e., difficulties in determining whether or not the biographical event actually took place). The internal negotiation regarding the validity of the insight had implications for the patient's therapeutic process; extended integration therapy (described below) was required to enable at least partial resolution of the issue. This revelation occurred during a psilocybin session in a clinical trial in which a medium-high dose of psilocybin was administered (25 mg), alongside therapeutic support from a psychiatrist and psychologist. The session content focused mainly on a confrontation with a cruel creature which the participant took to represent one of his parents. As the battle with the monster subsided, the participant had an experience in which he felt himself to be an infant whose parent was attempting to smother him with a pillow: “It's my mum. … It feels like a pillow over me. No it couldn't have been that. Did you really not want me that badly?”

This initial revelation was then scrutinised by the participant thoroughly after the effects of psilocybin had subsided. Immediately after the session, the participant expressed the emotional intensity of the contents experienced and questioned whether the experience corresponded to a true memory. In the two months after the session, communications from the participant to the study team revealed that he was experiencing remission from depression and was relatively untroubled by the “memory”. However, six months after the session, the participant described that the depression had returned and reported being now, understandably, quite preoccupied and confused about whether the suffocation event had happened to him:

So it's [about] trying to move on, it's all very well people saying “it's all totally symbolic”, I don't think it is. When it happened it felt more real than the here and now … I do think that something must have happened. Something pretty severe in my childhood for this treatment to take me back there … But what do you do with it? Do you just deal with it?

This quote illustrates ethical issues which can accompany psychedelic-induced biographical insights in therapeutic contexts by displaying the participant's distress concerning the revelation of a biographical event, facilitated by psilocybin intake, which might or might not have taken place. The participant describes thinking that the psilocybin session had probably revealed to him a previously unknown traumatic event, and that the alternative explanation—that the noetic character of psychedelic experience had apportioned misplaced validity to a symbolic scene—is less convincing. He accentuates that the experience “felt more real than the here and now”.

Due to the participant's ongoing distress about the veracity of the scene, the study team referred him for 10 sessions of extended “Integration” with a specialist therapist. Integration refers to sessions performed in a therapeutic or counselling format focusing on the contents that emerge during a psychedelic session. The participant reported that these integration sessions allowed him to reframe his experience in a new way. Regarding the process of integration, the participant said the following:

That information [revealed in the smothering scene during the session] was so new and quite useful, you need to make sense of it. Integration for me is as important as set and setting. It's important getting to the point of having revelations. Regardless of whether it's true or not, that information needs to be understood by your consciousness, it's going to be rattling around: did it happen, didn't it happen, and that's not the point at all. This message has come along, [so] what does it mean?

Integration may provide support in the process of becoming aware of new information, in which the role of a guide or therapist is crucial. From this perspective, knowledge gained during psychedelic experiences may be subjected to a process of intersubjective *mediation* (see Varela & Shear, 1999), which in this context may have a significant role for therapeutic outcomes. We will return to how this process may be inserted within practices that enhance the process of validation and mediation of psychedelic experiences in the last section.

While the process of *integration* may be helpful with dilemmas of validity in individual therapeutic process in Western contexts, other challenges may arise in other scenarios in which the mediation process is more strongly directed. We now turn to our second example, in which the psychedelic experience occurs in a neo-shamanic centre.

### Case 2. Revelations in neo-shamanic contexts

In this example we refer to the ritual use of ayahuasca as administered in the numerous neo-shamanic centres that have recently appeared on the edge of the Peruvian Amazon metropolitan areas in the context of the emergence of “shamanic tourism” (Labate & Jungaberble, 2011). These reception centres are most often based on the partnership of Westerners and mestizo or indigenous locals, and offer the opportunity to participate in ritual activities to an international clientele, inspired more or less freely by the practices of the Peruvian mestizo shamanism, including the ritualised use of the psychedelic brew ayahuasca.

Based on an ethnographic survey conducted between 2008 and 2013 in Takiwasi, a well-known centre of the area, Dupuis (2021a) has proposed that contents of ayahuasca experiences (and associated revelations) arise from an interaction between the psychopharmacological effects induced by ayahuasca and the social context surrounding its use (verbal exchanges, ritual interactions, etc.), which strongly influence the phenomenological features of the psychedelic experience. During these retreats, visual and auditory imagery perceived by the participants are indeed progressively perceived as acts of communication of supernatural beings (i.e., “voices” and “visions”) consistent with the cosmology of the institution. Cultural background and social interactions result here in a “socialisation of hallucinations” (Dupuis, 2021a), a social learning process, not only shaping the relationship to the psychedelic experience, but also its very phenomenology.

The visitors of this centre attend two-week retreats for the purposes of “personal development” during which they usually work with personal issues. Usually, approximately 15 visitors attend each retreat, which consists of introductory lectures, ritual activities, the ingestion of emetic plants (including ayahuasca) and post-session discussions. In the sessions before and after the ayahuasca ceremonies, participants are introduced to the specific cosmovision of the centre, which combines neo-shamanic, psychotherapeutic, biomedical and folk Catholicism elements. Within this framework, the psychological suffering of participants is frequently understood as resulting from demonic influence or possession (i.e., “infestation”). Retreat participants usually report the perception of demonic evil beings, which they most often describe as fighting against protective entities such as spirits of nature (e.g., the spirit of ayahuasca) or entities of the Christian pantheon. The following are two reports from visitors attending these retreats:

There were all these demons parasitising me inside, but I saw the ayahuasca that was chasing them, like a lot of little bright snakes inside my body that were circulating and cleaning all that up. … Later I saw ayahuasca. She was a kind of woman with a snake-like lower body, showing me how the demons got in, what I had done, and therefore what I had to do to stop them from entering.

At one point I had a vision with the archangel Saint Michael who was piercing a demon with his sword, as in the religious images. Later I felt the presence of Christ, who looked behind my back, where chains were hung connected to a cage. I saw my demons laughing because I had to drag my cage to move forward in life. They jerked me around all the time, like they were raping me. … When Christ saw the chains and the cage, he said it had nothing to do here and kicked to kick it all out.

These visionary narratives vividly reflect the cosmology and the specific *etiological theory* of the institution, which specifies that the source of illness occurs as a consequence of possession by demonic beings. Following the initial instances in which ayahuasca is taken, retreat participants may experience anxiety, uncertainty and misunderstanding, which are expressed in the post-session discussions. During these discussion groups, it is frequently suggested to participants (by fellow participants and ritual specialists or facilitators) that some aspects of their experience reveal the presence of demonic entities (“infestation diagnosis”) or benevolent agents (nature spirits, Catholic pantheon beings). Group discussions serve here as practices of mediation in which participants reframe psychological or somatic issues by adopting specific cultural motifs of “possession”, which are then experienced during subsequent ayahuasca sessions.

During and after the ayahuasca sessions, participants at this centre may ultimately be advised to adopt practices to free themselves from parasitic influences and the corresponding distress associated with them. These techniques involve contracting relationships with supernatural protective entities (plant's spirits, ancestors, Catholic pantheon entities) through prayer, participation in Catholic masses or the use of Catholic artefacts and practices (e.g., exorcism rituals, baptism). The discovery of the demonic influence will thus lead some participants to a (re)conversion to Catholicism and religious practice, that is perceived as a means of protecting themselves from demonic influences.

Taking on this framework may be useful for participants, as externalising the source of distress can have significant therapeutic value. “Externalising the problem” is a tool widely used in mainstream talk therapy (e.g., White, 2007) to provide people with a sense of agency, validation, hope, and improved self-esteem. Here the tension between pragmatic and ethical considerations is rendered clearly. The capacity of psychedelics to facilitate the appropriation of an etiological or cosmological theory by means of their tangible verification during the visionary experience acts indeed as a double-edged sword. This acts as a possible vector of therapeutic effectiveness, but also as a possible spring of religious conversion that remains largely implicit and unconscious (Dupuis, 2018a, 2021b), and which may consequently not be fully consented to. This case is an example in which heavily directed mediation processes suggest how deeply the ideology of a context may implicitly influence the psychedelic experience and its after-effects.

### Case 3. “Metaphysical” revelations in research contexts

In addition to revelations occurring in therapeutic and neo-shamanic contexts, psychedelic revelations featuring “metaphysical” themes (i.e., concerning the nature of reality) may occur in contexts associated with consciousness research. The validation of these experiences poses challenges to consciousness researchers attempting to determine invariant features of psychedelic phenomenology, as well as to research subjects attempting to make sense of their experiences. Importantly, psychedelic use may result in changes in worldviews which persist long after a session is over (Timmermann et al., 2021). We have captured some of these “metaphysical experiences” in some of our studies employing Dimethyltryptamine (DMT) at Imperial College London (see Timmermann et al., 2018). The following quote from a participant was obtained in an interview immediately after the DMT experience took place:

I felt the presence of this alien substance. This green, gooey alien substance which is not organic. … Everything is silicon based. Stuff which once was gooey, but now is fossilised. … This fossilised gooey stuff … is what reality is. The forests you see on planet Earth… that's a beautiful simulation … There are humans on these shelves, in very tight spaces. That's how I felt. This very, very compressed space. … So, finally, I felt, ah, I got to know what was in that forest … Every human body is just on one shelf, and there is another human on another shelf. And that's reality. The reason why we are not suffering is because machines are generating this beautiful reality for us.

For some participants, DMT-induced insights may last long after a session has ended and may inform subsequent experiences. The following quote by another participant from the same studies illustrates how these insights may have lasting impressions:

Entering a parallel world, parallel universe. Maybe what we are in the future. Maybe what we access is our evolution. This is informed by previous experiences, it's also informed by what I’ve read, but it is first-hand experience.

While participants may ask themselves: “Did I actually gain some important information regarding the nature of reality?”, researchers might question the validity of the reports of participants and what we can learn from psychedelic-phenomenology and ask “did the participant actually experience that?”. Putting it differently, the researcher might question the validity of first-person reports as reliable data to understand the effects of these compounds on human consciousness.

Among the common biases associated with first-person reports in consciousness research, psychedelics may be particularly prone to induce a confusion between experience and representation. Phenomenological research highlights that experiencers’ expectations (which are shaped by their specific cultural milieu), aided by language, may play a pivotal role in the recollection of an experience (Petitmengin, 2006). Specifically, participants are mostly aware of things that are consistent with their representations and beliefs, and, in the process of recollection, the original experience may be deformed through the influence of these beliefs and representations. Furthermore, psychedelic experiences may be especially susceptible to deformation, primarily due to their ineffable quality (i.e., the participant's inability to express aspects of the experience in language; Pahnke & Richards, 1970). Furthermore, popular “memes” abound in the cultural milieu associated with psychedelic drug use, and these themes could be having a significant effect in the contents experimented and recalled during psychedelic experiences.

Some preliminary data we have gathered in ritual settings in Europe reveal that psychedelic experiences taking place in modern ceremonies (many of them of a secular kind) may significantly alter beliefs regarding the nature of consciousness and reality. Specifically, results from a prospective survey reveal that following a retreat involving a psychedelic, respondents were more prone to endorse beliefs consistent with notions of the existence of separate realities, mind–body dualism and fatalistic determinism, and these changes remained significant for at least 6 months. Importantly, we found that the best mediator for these changes in beliefs was the degree to which participants felt *positive emotional synchrony* (i.e., the experience of sharing feelings of positive affect with a group; see Páez et al., 2015, for details on this construct). This effect was moderated by respondents’ pre-session scores of peer conformity. These results highlight how the role of intersubjective factors and social attitudes result in long-term changes in worldviews during psychedelic experiences (Timmermann et al., 2021). While our interest is not to debate the actual ontological validity of such metaphysical insights, we see the value in a gentle, open space for participants to examine and revisit these insights in the post-session period. This is especially relevant considering that these changes in supernatural beliefs were significantly associated with increases in well-being up to 6 months after the retreat took place, further stressing pragmatic and ethical tensions—a double-edged sword.

## Contextual embeddedness of mediation and validation

**Figure 2.**
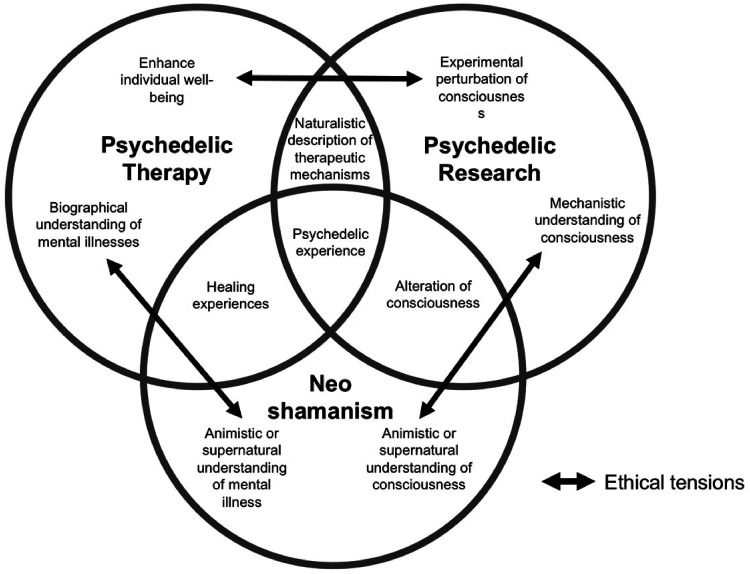
Examples of institutional aims for a psychedelic session.

*Note*. Overlaps of aims for a psychedelic session between three different contexts in which psychedelics are used. Sources of ethical tensions can be found between specific diverging aims. The aims presented are not meant to be exhaustive, but simply illustrative.

As seen in the examples above, processes of mediation and validation appear to have a strong embeddedness within the larger context in which revelations occur, regardless of their subjectively felt unmediated character. Furthermore, issues of validation and mediation of psychedelic experiences are further exacerbated by the fact that these experiences have not been legitimised in the Global North (psychedelics are illegal in most Western countries), which renders the instances for mediation highly unstable. Different institutions, for example, religious traditions and the medical or scientific field, are playing a competing role in the construction, legitimisation and maintenance of new and hybrid practices that are multiplying around the use of ayahuasca (Dupuis, 2018b). This is especially relevant considering that the noetic quality may act as a catalyst for each of these institutions’ frameworks to be validated and legitimised through users’ personal experiences. Furthermore, each of these institutions have their own aims and ethical issues are expected to arise when these aims are divergent. These institutional aims represent the *staging* through which psychedelic-induced revelations are expressed and played out during a psychedelic session (Figure 2).

## Towards psychedelic apprenticeship: Frameworks for grounded practices

Considering that the subtleties of dealing with psychedelic experiences will continue to present challenges to facilitators and providers, we outline a *coping* framework to understand issues of mediation and validation. These strategies are fundamentally grounded in forms of *apprenticeship*, which aims to foster acts of becoming aware of users’ own mental states. As noted by Depraz et al. (2003), characterising practices involving the careful examination of experience (i.e., introspection) may be elusive because their complexity may be masked by the apparent ease of access we have to our experience:

Introspection is difficult, it demands an apprenticeship, requires progressive development of a genuine expertise. The greatest difficulty lies in the fact that this technicality is masked, that it can pass unnoticed due to the apparent ease with which it is possible to obtain a minimum amount of knowledge about our states of mind, our thought processes, our emotions. (Depraz et al., 2003, p. 154)

Once an experience of insight or revelation has taken place within a psychedelic session, then the users (or researchers) may be left with the following questions: Are these experiences real? Did I really re-experience a repressed memory which actually took place? Did I really meet usually invisible beings such as demons, ancestors and plant spirits? Did the participant really experience that, or are the contents symbolising some emotional truth instead? Could it be simply experiential “noise”? These are questions which do not have straightforward answers, and we do not intend to try to answer them here. We wish, however, to outline (1) the subtle ways in which these processes of intersubjective mediation and validation may take place; (2) the role that guides and facilitators have in this regard; and (3) examples of practices and methodologies that may be useful in this process. These three points can be considered as being part of a process of *apprenticeship* (Depraz et al., 2003), which can be useful for users, facilitators and the larger community of researchers and therapists seeking to enhance awareness regarding the nuances of psychedelic experiences and therapeutic processes.

### Intersubjective mediation

Insights initially encountered within a private domain of experience are also embedded in complex social networks susceptible to intersubjective mediation and validation (Depraz et al., 2003). The challenges surrounding the validation of information obtained in a psychedelic session lie in the fact that the position within the social network through which the information is achieved is closer to a first-person position when compared to, for example, scientific insights gained through a method closer to a third-person position which is validated through a peer-review process practised by a community of researchers (and thus inserted in a complex social network). We see as imperative that methodologies dealing with issues of mediation of psychedelic experiences take into account the possibility of extending the process of validation of first-person psychedelic-induced insights into a broader intersubjective milieu beyond the sphere of private experience.

Acts of becoming aware are pervasive in human experience but can also be structured in such a way that they promote a certain amount of reflection. We consider this to be particularly important in situations in which gaining knowledge has an apparent unmediated (i.e., noetic) character. Contemplative practices are other examples of situations that undergo similar issues of validation, as in these practices, the central importance of first-person experience in the process of gaining knowledge is also pivotal. While the first step in validation of these experiences is always private and rooted in a process of *intuitive fulfilment* (see below and Depraz et al., 2003), by not subjecting these insights into broader contexts, the process of becoming aware may be entirely reduced to a detrimental form of individual validation, akin to solipsism (Depraz et al., 2003). As illustrated by the controversies surrounding repressed memories of sexual abuse or recovered memory therapy (Freud, 1897/1985; Hacking, 1995; Loftus, 1993), many psychotherapeutic practices aiming to foster self-knowledge through the use of introspective techniques and interactional devices (e.g., psychoanalysis and clinical hypnosis) have faced the risk of developing iatrogenic complications such as false memory syndrome. We argue that the process of psychedelic-induced insights may exacerbate some of these risks due to their ability to induce noetic feelings and increased levels of suggestibility (Carhart-Harris et al., 2015).

While psychedelic insights and revelations may feel to users as having an unmediated character, the process of validation can be viewed from a mediated and intersubjective perspective. For example, all three cases outlined above deal with issues which could be conceived as tensions between first and third-person validation. In Case 1, the participant experiences revelations that pertain to both his subjective experience (first-person) and to potentially real biographical information (verifiable from a third-person position). In Case 2, insights occur as part of transmission of specific motifs corresponding to the institution's cosmovision (third person) which are incorporated into the lived experiences and psychological life of retreat participants (first-person). In Case 3, participants’ psychedelic experiences (first-person) are validated by researchers (third person) collecting data at an immediate level, and also by a wider community of researchers. These examples stress the flexile nature that psychedelic-induced insights can take, regardless of their immediate and felt unmediated quality they have when they occur. Bringing awareness of methods to mediate these insights may be a crucial step to optimise psychological safety and efficacy, while also developing expertise in the process of facilitating psychedelic experiences in diverse contexts.

### Role of the facilitator: Empathic resonance

Expression and validation are processes which imply the insertion of the act of becoming aware of insights within a socially situated context (Varela & Shear, 1999). The process of validation usually takes place within an intersubjective continuum: it may occur with a first-, second- or third person position. From the first-person position, validation occurs via *intuitive fulfilment* (no expression necessarily taking place). The second-person position is the one we are interested in here, whereby validation takes place via *empathic resonance*. In this scenario, the process of expression and validation takes the form of an *apprenticeship*: a therapist, a spiritual facilitator or a guide, has undergone a similar process as the experiencer and thus can provide gradual orientation to the process of navigating this experiential domain and aid in assimilating these insights (Depraz et al., 2003).

In the case of psychedelic sessions inserted within a psychotherapeutic process (from the point of view of the experiencer), this process of training may take the form of sessions of *preparation* and *integration* in which there are recursive dynamics allowing for the process of achieving insights to begin before the actual moment when the session takes place. By fostering preparation, setting intentions, and expressing expectations before a session, as well as reflecting upon the experience in an open-ended fashion after the experience has taken place, the process of becoming aware, aided by psychedelic experiences can become rooted within the larger social, historical and cultural context in which the user, the guide and the knowledge on which the practice (therapeutic session, ritual, experiment) is based upon are all taken into account. While the notions of *preparation* and *integration* are becoming commonplace in different therapeutic frameworks for psychedelic therapy, we would like to stress here the central importance that a means of validation based on *empathic resonance* by an experienced guide/therapist has for this process, as this will affect crucial interventions before, during and after the session. From the point of view of the therapist, guide or facilitator, forms of apprenticeship include instances of therapeutic supervision by more experienced individuals as well as opportunities for learning associated with their own experiences involving psychedelics.

Within this framework, it may be important to consider that training of psychedelic therapists involves opportunities for them to have guided psychedelic experiences within therapeutic frameworks of mediation. Similarly, in the case of researchers investigating psychedelic phenomenology, a second-person position of *empathetic resonance* employing interview devices that deepen the process of phenomenological enquiry (see Petitmengin, 2006; Petitmengin et al., 2018) of their research participants beyond the use of psychometric scales will enable the wider scientific community to learn *more* regarding the invariants associated with the psychedelic state of consciousness. We believe that this second-person approach fosters opportunities to deepen psychedelic *knowing* for individuals and can guide the scientific community more widely.

## Specific methodologies and practices

### Detailed phenomenological inquiry

Some techniques may foster better ways to recollect and reflect upon specific experiences than others. A useful technique we have used in a research context (see Timmermann et al., 2019) is the microphenomenological interview (MPI) technique. The MPI consists of a disciplined approach to re-evoke and recollect past experiences through a mediated (i.e., second-person) approach. The process of mediation consists of techniques aimed to (1) stabilise the attention of the individual; (2) promote attention to turn from contents (*what*) to the processes (*how*) of experience; and (3) help participants divert the focus from generic to specific dimensions of experiential structures (see Petitmengin, 2006, for details). The MPI has been used in different research contexts aiming to determine the micro-structures of meditation (Przyrembel & Singer, 2018), intuition (Petitmengin-peugeot, 1999), mind-wandering (Petitmengin et al., 2017) and mood disorders (Depraz et al., 2017). We argue that elements from this technique may be applied in research and therapeutic contexts, both to address issues of determining invariant structures of psychedelic experiences in consciousness research, as well as for patients who could benefit from further recollection and exploration of instances of therapeutic insight (Davis et al., 2019) and *emotional breakthroughs* induced by psychedelics (Roseman et al., 2019). These could then provide important valuable therapeutic material to be used within patients’ broader therapeutic processes. Detailed and mediated phenomenological inquiry could allow users to *express more* and better by minimising the influence of previous knowledge on the recollection of the subject's experience (Depraz et al., 2003).

### The accept–connect–embody approach

The initial psilocybin for depression study at Imperial College, from which Case 1 was a part, provided the basis for the development of a clinical model, which was adopted in the second psilocybin for depression study. This model is called ACE, which stands for, “Accept, Connect, Embody” (Watts & Luoma, 2020). Based on Acceptance and Commitment Therapy (ACT; Hayes et al., 2006) and the Psychological Flexibility Model (PFM; Hayes et al., 2006), the model maps three key mechanisms of psychedelic therapy: acceptance of emotion, connection to meaning, and a level of processing that occurs in the whole body, rather than just in cognition. The ACE model can be applied in flexible ways and can suggest a way forward at times when a participant feels “stuck”, or at an impasse. Validation dilemmas, of the type discussed above, can lead to such an impasse: the participant is confused, the therapist-guide has no answers, and both can search for clarity about the objective truth as the only way to proceed. As this objective truth is elusive, it may be more productive to consider what is “helpful” rather than what is “true”—this is a foundational concept in contextual behavioural science, upon which ACT and the PFM are based. From a therapeutic perspective, the ACE model offers the methodology presented in Table 1 in cases of validation confusion, with suggestions here for ways to introduce the exercises to a patient (see Watts & Luoma, 2020 for details).

**Table 1.** ACE method for assisting a client at an impasse.

The following are suggestions for therapists dealing with patients and issues of validation confusion based on the ACE model (Watts & Luoma, 2020).
**A: Accept**
We could try to explore the experience deeper if we disengage from thoughts about whether it is true or not, just for now. If we let go of trying to find the answer for a while, we can look at what you saw/felt as valuable information in its own right, which could be helpful to you in your life, regardless of whether it actually happened. Are you willing to lie down, and revisit the scene you saw? If the scene/impression you saw/felt/thought were true, what emotion would be elicited? Can you let this emotion grow in you, and sit with it for a while. Where do you feel it in your body? Let the feeling grow, get bigger, and let me know what is happening and if you would like some support (ways of supporting each individual will have been discussed before the psychedelic session, e.g., hand holding, soothing words, music). If there is anger, shame, fear, disgust, etc., any feelings that feel really painful to bear, keep breathing into the places that you feel these feelings, and remember that painful feelings are an important guidance system. Stay with the feelings, keep breathing and remember that you are safe now. As the feeling develops, keep focusing on where you feel it in your body and notice if the sensations are changing. Keep breathing gently into the places you feel these sensations.
**C: Connect**
Now that you have been with these feelings, try to connect to your “observer self”. This the part of you who is always watching your experiences and doesn’t get damaged by them. A bit like the way the sky doesn’t get damaged by the weather; no matter how stormy, even the biggest flash of lightning doesn’t burn the sky. So, connect to that part of you that witnessed that scene or impression, and connect to the part of you that has just experienced these strong feelings in your body. Just take a few moments now to breathe, and listen to this piece of music, and just reflect on 1) Why do you think you saw that scene, what was it trying to teach you/show you? 2) What do you think these emotions and sensations you just experienced were trying to show you? What is the meaning here? 3) What does this all teach you about what really matters to you? Those painful feelings—what do they show you about what you need in order to feel safe or what you value.
(If the scene relates to an event in infancy): what would your observer self say to you as a child? Imagine you could visit that scene, what would you do or say to all the people involved? What does this tell you about the values you hold? You might like to consider some action which you can do, to honour these values you hold as an adult. Examples include: a ritual to honour the positive values that grew in them as a result of some of the painful feelings they have felt in their life; writing a letter to some of the individuals involved in the scene—their own infant self, a caregiver, etc. Such letters are recommended to be written for creative purposes, not to be sent—after all this exercise is exploration “as if”, there is no final judgement about whether anyone actually harmed them.
**E: Embody**
It may be that some individuals feel that these explorations are not satisfactory and they still wish to pursue the truth. Presumably, if it is possible to seek external verification, they will do so (e.g., by asking someone who was there at the time). If this is not possible, then they can engage in the following somatic enquiry exercise to obtain some sense of what feels true for them. This exercise involves listening to two pieces of music. Music choices are at the discretion of the therapist:
Lie down, and then I will play you the first piece of music. Bring your attention to your breath. Then, go back to that scene/impression you saw or felt. Feel whatever emotions come up. Then, hold the idea that it is true, that it really happened. Breathe, follow the music. Let whatever emotions and sensations develop. Just be there. Then you will listen to the second piece of music, going back to that scene, but this time holding in mind the idea that it did not happen, and is symbolic. (Conduct the exercise).
What happened in your body in the first and second piece of music? Do you get some “gut feeling”? Did it feel truer to you that it really happened or was it symbolic? This exercise cannot tell you which is true. It is intended to help you tune in to your own “inner intelligence” as a way of finding some understanding, and peace. Remember to hold any insights lightly.

### Accumulated know-how

We would also like to mention practices, such as those used by therapists, facilitators and mestizo/indigenous shamans, which have formed valuable psychedelic know-how employed to this day in psychedelic use. These have not emerged from a structured theoretical framework defined a priori, but rather have been developed based on the embodied practice of shamans, psychotherapists, researchers and experiencers (see Beyer, 2011; Grof, 1975; Johnson et al., 2008; Meckel Fisher, 2015; Naranjo, 1974; Richards, 2016; Shanon, 2005; Shulgin & Shulgin, 1995, for examples). Among these practices we highlight the use of structured instances surrounding the psychedelic experience (e.g., preparation and integration sessions), psychological support during dosing sessions, the use of the ritual setting and tools, such as music and perfumes, to foster safety and enhance therapeutic intentions and detailed attention to users’ experience (Dupuis, 2021a). All these instances may provide opportunities for experiences to be expressed, reflected upon and validated in an intersubjective way, in which the role of the guide or facilitator is consistent with the notion of *empathic resonance* we mention above. We believe that this *know-how*, resonant with the term of *apprenticeship* outlined above, has yielded important foundations to deal with issues of mediation and validation.

**Figure 3.**
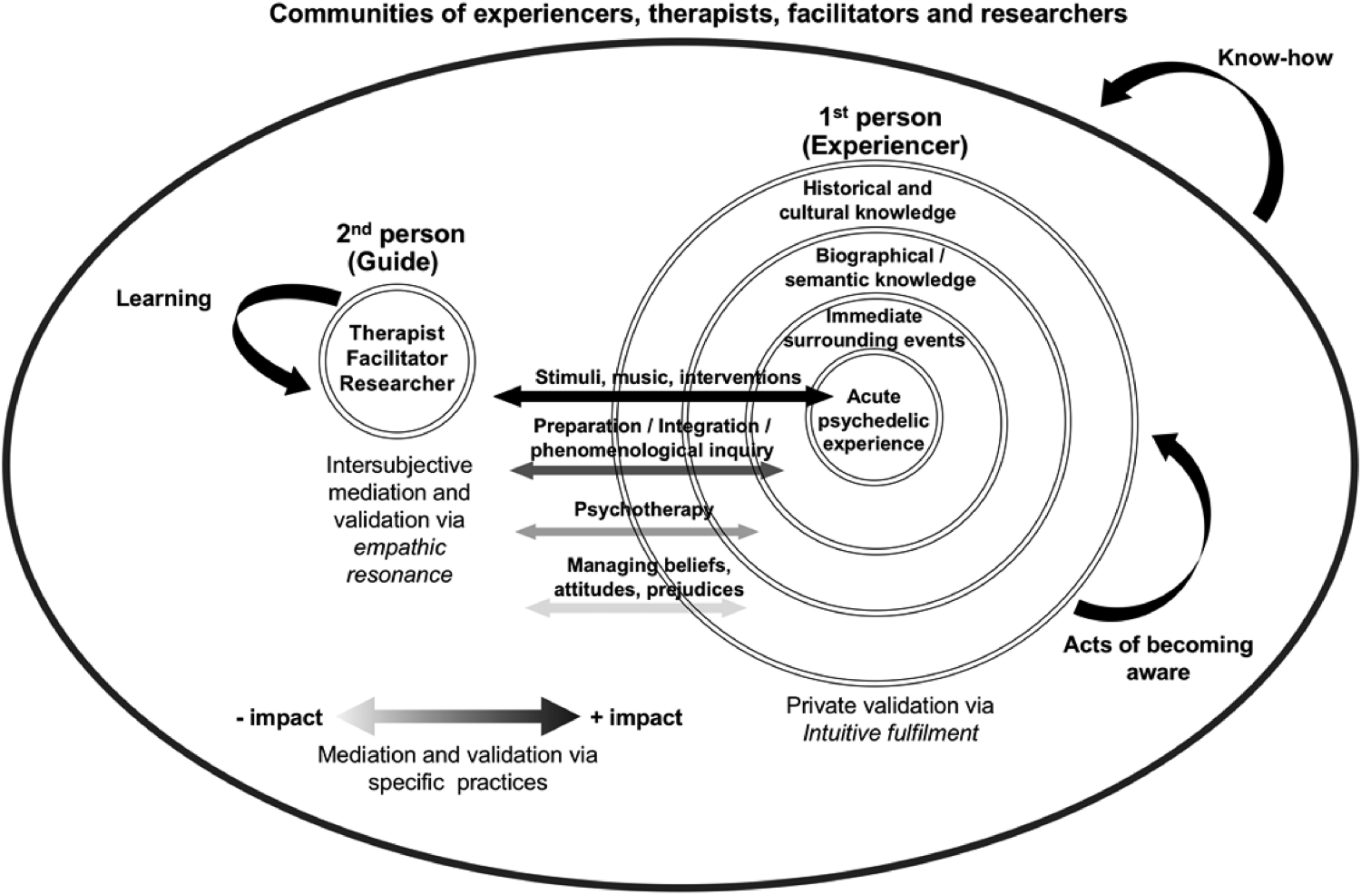
A framework for psychedelic apprenticeship.

*Note*. Modification of Figure 1, illustrating the experiencer (concentric circles), the guide (small circle), examples of interventions currently employed in some forms of psychedelic use as forms of mediation and validation and their bi-directional impact (bi-directional arrows). Also illustrated are the social positions of each of these actors and their insertion within a broader community. Finally, the ensuing dynamics of these practices promote instances of becoming aware for experiencers, learning for guides and the development of know-how for the wider community (curved arrows).

Under this view of *apprenticeship*, acts of becoming aware are not constrained to the experiencer, but also involve the guide, as the evaluation and results from their intervention will result in the development of learning instances, which are part of the construction of an embodied “know-how” rooted in experiential guiding and facilitating of sessions or therapeutic processes. Under the specific context of psychedelic therapy in the West, we consider these forms of experiential and embodied learning a crucial element in the development of therapeutic abilities and validation of phenomenological data for research purposes. These instances of learning can be further promoted through the use of their own process of development of therapeutic abilities and continuous instances of supervision, among many others. In wider temporal and spatial scales, this recursive process of learning involving experiencers and facilitators may provide the wider communities of psychedelic experiencers, facilitators and researchers instances to develop knowledge (a “know-how”) regarding psychedelic experiences and therapeutic processes. Using this framework of apprenticeship, further practices and methods can be developed and formalised into specific interventions which foster recursive forms of experiential inquiry and acts of reflection (Figure 3).

## Conclusion: Ethical know-how for psychedelic practices

The act of knowing during psychedelic experiences can be thought of as analogous to other acts of becoming aware which depend (at least initially) on intuitive (or unmediated) evidence. Intuitive acts carry with them a sense of novelty, which is lived as an emergence of content which is unpredictable and thus presents a discontinuity in experience. This moment of intuitive fulfilment can be optimised by a double movement consisting of both *passive acceptance* as well as the *holding the grasp* of such an experience in a light-handed fashion so that instances are generated which may allow for contents to have an open-ended aspect to them (Depraz et al., 2003). We have proposed in this article a framework which may aid in the process of mediation and validation of psychedelic insights after outlining some specific issues that may arise in different contexts. Our proposed framework aims to present the characteristics of the context, the role of the facilitator and specific methodologies which can aid in the development of a gentle touch when it comes to psychedelic insights. We aim to do this not for the sole purpose of dealing with issues of validation, but also for enhancing opportunities and developing practices required to navigate the subtleties of the psychedelic experience. Under this notion we echo the sentiment that, like contemplative practices, psychedelic experiences require a degree of discipline embedded in forms of apprenticeship to manage these risks during the process of transformation of the subject that engages with them.

This process of apprenticeship is intimately linked to a notion of ethics which (for the most part) denotes ethical action as the result of an embodied practice (i.e., ethical know-how; Varela, 1999), rather than a set of clearly delineated rules determined a priori. While it can be said that safety aspects associated with ingestion of psychedelics do require a set of principles which have been also prescribed a priori (see Johnson et al., 2008, for an example), it is important to note that these principles have been in large part derived from the embodied practice involving psychedelics both within and outside sanctioned environments and the assimilation of these precepts as guidelines for good practices are consistent with this notion of ethical know-how or apprenticeship. From the point of view of the psychedelic experiencer, these precepts will help, but will not carry the bulk of the chore at hand, as evidence is consistently showing that it is through experience that the desired therapeutic effects associated with psychedelics occur (Davis et al., 2019; Garcia-Romeu et al., 2014; Roseman et al., 2018). It is in the experience where the epistemic challenges we outline above will occur, and it is in a progressive engagement with the process of experience that the subject will eventually learn to navigate the subtleties of the psychedelic space. We argue that this process is recursive and can be aided by contextual devices and practices, some of which we have outlined above. It is in this process that experiencers will become acquainted with the dynamics of knowing and “letting-go”, which appear to be so crucial not only for psychedelic experiences and psychotherapy processes, but also in the contemplative traditions which have inspired this notion of ethical know-how (Varela, 1999).

To conclude, we argue that forms of psychedelic apprenticeship are required to be grounded on experiential forms of “know-how”, which foster the development of devices that allow recursive forms of experiential inquiry. This is especially relevant considering the illegality of psychedelic use in many Western settings, the increasing demand for psychedelic therapy (see Nutt et al., 2020, for an up-to-date overview) and the exponential increase in interest associated with psychedelics in current times. We expect that the scaling up of psychedelic use will highlight the ethical issues we have outlined here, while also underscoring the value of disciplined inquiry into the subtleties of these practices and experiences. It is possible that psychedelic practices may find inspiration in contemplative traditions which have historically embedded their practices, to develop gentle ways to engage with insights that support processes of learning, development and transformation:

Talk alone will certainly not suffice to engender spontaneous non-egocentric concerns and ethically developed persons. Even more than experiences of insight, words and concepts can easily be grasped at, taken as ground, and woven into a cloak of egohood. Teachers in all contemplative traditions warn against taking fixed views and concepts as reality. We simply cannot overlook the need for some form of sustained practice or *pratique de transformation de sujet*. (Varela, 1999, pp. 74–75)

## Notes

1. We use the term “neo-shamanic” in a descriptive way to refer to contexts which employ practices and discourses that integrate indigenous practices and Western psychotherapeutic elements (Scuro & Rodd, 2018).
